# *Bacillus amyloliquefaciens* Rescues Glycyrrhizic Acid Loss Under Drought Stress in *Glycyrrhiza uralensis* by Activating the Jasmonic Acid Pathway

**DOI:** 10.3389/fmicb.2021.798525

**Published:** 2022-03-16

**Authors:** Liang Yue, Constantine Uwaremwe, Yuan Tian, Yang Liu, Xia Zhao, Qin Zhou, Yun Wang, Yubao Zhang, Bailong Liu, Zengtuan Cui, Chengchao Dun, Ruoyu Wang

**Affiliations:** ^1^Key Laboratory of Stress Physiology and Ecology in Cold and Arid Regions of Gansu Province, Northwest Institute of Eco-Environment and Resources, Chinese Academy of Sciences, Lanzhou, China; ^2^Gansu Gaolan Field Scientific Observation and Research Station for Agricultural Ecosystem, Northwest Institute of Eco-Environment and Resources, Chinese Academy of Sciences, Lanzhou, China; ^3^University of Chinese Academy of Sciences, Beijing, China; ^4^CAS Key Laboratory of Tropical Forest Ecology, Xishuangbanna Tropical Botanical Garden, Chinese Academy of Sciences, Mengla, China; ^5^Key Laboratory of Desert and Desertification, Northwest Institute of Eco-Environment and Resources, Chinese Academy of Sciences, Lanzhou, China; ^6^Gansu Institute for Drug Control, Lanzhou, China; ^7^The General Station of Construction and Protection for The Cultivated Land and Quality of Gansu Province, Lanzhou, China; ^8^School of Management, Lanzhou University, Lanzhou, China

**Keywords:** PGPR, *Glycyrrhiza uralensis*, glycyrrhizic acid (GA), drought, jasmonic acid (JA)

## Abstract

Drought is a major factor limiting the production of the perennial medicinal plant *Glycyrrhiza uralensis* Fisch. (Fabaceae) in Northwest China. In this study, 1-year-old potted plants were inoculated with the strain *Bacillus amyloliquefaciens* FZB42, using a gradient of concentrations (CFU), to test for microbe-induced host tolerance to drought condition treatments in a greenhouse experiment. At the concentration of 10^8^ CFU ml^–1^, FZB42 had significant growth-promoting effect on *G. uralensis*: the root biomass was 1.52, 0.84, 0.94, and 0.38 times that under normal watering and mild, moderate, and severe drought stress conditions, respectively. Under moderate drought, the positive impact of FZB42 on *G. uralensis* growth was most pronounced, with both developing axial and lateral roots strongly associated with indoleacetic acid (IAA) accumulation. An untargeted metabolomic analysis and physiological measurements of mature roots revealed that FZB42 improved the antioxidant system of *G. uralensis* through the accumulation of proline and sucrose, two osmotic adjustment solutes, and by promoting catalase (CAT) activity under moderate drought stress. Furthermore, significantly higher levels of total flavonoids, liquiritin, and glycyrrhizic acid (GA), the pharmacologically active substances of *G. uralensis*, were found in the roots of inoculated plants after FZB42 inoculation under all imposed drought conditions. The jasmonic acid (JA) content, which is closely related to plant defense responses and secondary metabolites’ production, was greatly increased in roots after the bacterial inoculations, indicating that FZB42 activated the JA pathway. Taken together, our results demonstrate that inoculation with FZB42 alleviates the losses in production and pharmacological metabolites of *G. uralensis* caused by drought *via* the JA pathway’s activation. These results provide a developed prospect of a microbial agent to improve the yield and quality of medical plants in arid and semi-arid regions.

## Introduction

Licorice (*Glycyrrhiza uralensis* Fisch.) is an “essential herbal medicine” in China, whose roots harbor plentiful pharmacologically active substances, such as saponins and flavonoids (Schrofelbauer et al., 2009). Glycyrrhizic acid (GA), the most bioactive component of licorice root, is a triterpenoid saponin that has been extensively studied not only for its multiple health benefits, namely, its anti-viral, anti-inflammatory, and anti-cancer effects, but also other pharmacological activities (Lin, 2003; Matsumoto et al., 2013; Su et al., 2017). Moreover, GA can be utilized as a multifunctional drug carrier (Selyutina and Polyakov, 2019). Another bioactive component is liquiritin (LIQ), it being the predominant flavonoid in licorice root and known to have a variety of pharmacological activities, including anti-inflammatory, antitussive, anti-asthmatic, analgesic, anti-cancer, and neuroprotective effects (Liu Z. et al., 2020).

Wild *G. uralensis* plants, in populations distributed in arid and semi-arid regions of East Asia including Northwest China, vary in their tolerance to abiotic stress factors, including drought (Pan et al., 2006). Due to dwindling natural resources, now scarce, and concern over vegetation degradation, wild *G. uralensis* individuals are no longer the main source sustaining the market supply of this medicinal plant. Accordingly, to meet rising demand for it, cultivated *G. uralensis* has since become the paramount source sold in the current market; however, compared with the traits of wild stocks, both adequate soil fertility and fertilizer supplementation have probably weakened its tolerance to abiotic stresses (Liu et al., 2014; Li et al., 2016). Improving the ability of *G. uralensis* to withstand droughts in arid and semi-arid region where it is now *G. uralensis* mainly cultivated is imperative for securing the production and satisfying its burgeoning market demand worldwide.

Drought stress conditions prevail in drylands, including arid and semi-arid regions, where the scarcity of water limits the distribution and survival of plants and even local crop production (Lu and Zhang, 1998; Dekankova et al., 2004). A recent report concluded that half (50%) of all crop losses are now caused by abiotic stresses, most of which is from drought (10%) and heat (20%) (Zia et al., 2021). In plants incurring drought stress, typically their photosynthetic machinery is impaired and photorespiration increases, thus disrupting their cells’ homeostasis; this then elicits the abundant generation of reactive oxygen species (ROS), the hallmark of and foremost plant response to stress. These ROS can cause much damage because they are highly reactive and toxic to proteins, lipids, and nucleic acids, which, if not dealt with by plants, eventually prolongs cellular damage and culminates in death (Moller et al., 2007). Plants under such stress will usually experience many metabolic, physiological, and biochemical changes. Organic solutes, however, such as soluble sugar, soluble protein, proline, and other low-molecular-weight metabolites, play key roles in how plants adjust to stress (Casanovas et al., 2002). Furthermore, antioxidant enzymes, such as superoxide, dismutase (SOD), peroxidase (POD), and catalase (CAT), can scavenge for ROS in plants (Zhu et al., 2009).

Plant-growth-promoting rhizobacteria (PGPR) can enhance plant growth directly, by attaching themselves onto roots, a feat possible because of their colonizing and biofilm-forming abilities (Bhattacharyya and Jha, 2012). In particular, PGPR could do more to help plants growing under drought conditions in several ways: optimizing the root environment and its water-absorbing capacity, accumulating antioxidant and osmolytes, and modifying phytohormones—the latter being a key player in plant defense responses (Naseem and Bano, 2014; Cohen et al., 2015; Poveda, 2020). For instance, the jasmonic acid (JA)/ethylene (ET) pathway usually involved in plant resistance can be induced by PGPR (Barnawal et al., 2017; Vries et al., 2020). For example, *Pseudomonas putida* H-2-3 increased the JA concentration and augmented antioxidant activity to enhance soybean (*Glycine max*. L. cv. Taekwang) plants’ growth under saline and drought conditions (Kang et al., 2014).

*Bacillus* is one of the predominant genera of PGPR. It shows their plant-promoting effect as a complex mix of modes of action: produce phytohormones with beneficial effects for plant growth and tolerance; produce volatile organic compounds (VOCs) capable of modulating growth and inducing resistance in plant; and active systemic plant responses involving phenolic compounds, genetic and structural modifications, plant resistance activators, and the activation of enzymatic weapons (Hashem et al., 2019; Poveda, 2020, 2021; Poveda and Gonzalez-Andres, 2021). Our research showed that the strain *Bacillus amyloliquefaciens* FZB42, a typical PGPR, is capable of conferring greater growth and stress tolerance to *Arabidopsis* through rhizosphere inoculation and volatiles emitted from the JA pathway (Hao et al., 2016; Liu et al., 2017; Lu et al., 2018; Liu S. et al., 2020). In addition, PGPR inoculations can also increase the content of secondary metabolites in host plants. Zhao et al. (2016) found that *B. amyloliquefaciens* GB03 stimulated accumulation of secondary metabolites in *C. pilosula*. Moreover, it was reported that pennyroyal (*Mentha pulegium* L.) reduced the damage to its physio-biochemical characteristics and production of secondary metabolites normally caused by drought (Asghari et al., 2020).

In this study, the effects of *B. amyloliquefaciens* FZB42 inoculation for improving *G. uralensis* tolerance to a range of realistic drought conditions were experimentally investigated. We measured plant physiological traits and carried out an untargeted metabolomic analysis of the root organ to elucidate bacterial-induced drought tolerance in *G. uralensis* mediated by osmotic solutes, antioxidase, and phytohormones, and the secondary metabolites GA and LIQ. Meanwhile, plant defense hormone JA plays a critical role in FZB42-induced drought tolerance of *G. uralensis*. The purpose is to provide a better understanding about the significant effect and mechanism of FZB42 on alleviating the adverse effect caused by drought stress on *G. uralensis*.

## Materials and Methods

### Plants and Bacteria

One-year-old cultivated *G. uralensis* plants were collected from a single location in Yuzhong County, Gansu Province, China (36°17″ N 104°33″ E), a typical loess hilly region; mean annual average temperature and annual precipitation were 6.9°C and 350 mm, respectively. The soil mainly consists of subalpine meadow soil and gray cinnamon soil (Lv et al., 2020; Wei et al., 2021).

*Bacillus amyloliquefaciens* FZB42 deposited as strain 10A6 in the culture collection at the Bacillus Genetic Stock Center (BGSC). The FZB42 strain was cultured overnight, on Luria–Bertani liquid medium, at 37°C with shaking (200 rpm). Then, its cells were obtained by centrifugation (10,000 × *g* for 6 min) and re-suspended in sterile water to yield four concentrations, 1.0 × 10^7^, 10^8^, and 10^9^ CFU ml^–1^, for use as inocula in the experiment.

### Plant-Growing Conditions and Treatments (Drought and Plant-Growth-Promoting Rhizobacteria)

The factorial pot experiment was conducted in a greenhouse at the Gansu Gaolan Field Scientific Observation and Research Station (36°13″ N 103°47″ E), in Lanzhou, China. The mean day and night temperatures were, respectively, 28 and 16°C, with a light/dark cycle of 14 h:10 h. On April 30, 2019, every *G. uralensis* plant was transplanted into a pot (30-cm diameter × 35-cm depth; 480 plants in total), each containing 20 kg of soil, which had been taken from the surface layer (0–30 cm depth) of a field at the station. The pH, C/N, TP, and TK of this soil was 8.54, 25.35, 1.00, and 19.13, respectively.

The experiment used a split plot design to test the effects of two crossed treatment factors (drought and inoculation), each with four levels. The drought conditions consisted of a (i) normal watering, that is, plants were grown in soil with the field water capacity (FWC) maintained between 60 and 65%, and likewise; (ii) mild drought stress, with an FWC of 55–60%; (iii) moderate drought stress, with an FWC of 45–50%; and (iv) severe drought stress, with an FWC of 35–40%. Then, in each of these four drought groups, their plants were rhizosphere inoculated with the FZB42 bacteria suspension at concentrations of 0, 1.0 × 10^7^, 10^8^, or 10^9^ CFU ml^–1^. In this experiment, the volume of water required to maintain the pot-level field capacity of each drought treatment corresponded to the amount drawn from soil by plants in previous days, assessed using the weighing method (i.e., weighing and watering the pots every 5 days). At 2 months post-transplanting, for the inoculations, 1,000 ml of the bacteria suspension for a given CFU was applied to the soil around each plant’s main root (∼10 cm); the non-inoculated plants (CFU = 0) received the same volume of water by similar methods. In addition, the greenhouse was covered with a transparent plastic canopy to avoid interference from rain.

Three months after imposing the drought treatments, all the experimental plants were removed from their potted soil and separated into shoot and root parts using shears, cleaned with distilled water, then immediately flash frozen in liquid nitrogen and stored at −80°C to preserve the ROS and antioxidant components and prevent lipid peroxidation, for their use in the later metabolomic analysis. Each treatment combination had 30 replicates.

### Plant Growth Response Variables and Biomass

At the experiment’s end—before removing them from their pots—the plants’ height, basal stem diameter, and the total biomass in each treatment combination were measured. Plant height was measured from the bottom of the stem to the terminal bud of the main stem. For basal diameter, the stem diameter of a plant was measured with a digital Vernier caliper at the soil surface. To determine biomass, plants were cleaned and divided into shoot and root tissues (see above), and these parts were weighed on an electronic balance (YP2001B, Lichen Tech, Nanjing, China).

### Osmotic-Adjustment Solutes and Antioxidase Activity

The proline content in the plant shoots (stem + leaves) and roots was determined according to the method described previously (Bates et al., 1973). Briefly, 0.5 g of fresh leaves was frozen in liquid nitrogen, homogenized by vortex in 3% (w/v) sulfosalicylic acid (Merck KGaA, Hamburg, Germany) in 2-ml microtubes, and immediately centrifuged at 10,000 × *g* for 5 min (Eppendorf 5415C Centrifuge, Hamburg, Germany); the pellet was then discarded. Next, 1 ml of supernatant was taken and mixed with a solution of 1.25 g ninhidrin (Merck KGaA, Hamburg, Germany) dissolved in 30 ml of acetic acid (Merck KGaA, Hamburg, Germany) and then mixed with 20 ml of 6 M phosphoric acid (Merck KGaA, Hamburg, Germany) for analysis. Its absorbance was measured immediately, at 520 nm, using a spectrometer (DU 530, Beckman Coulter, Brea, CA, United States) at room temperature (20 ± 2°C); the calibration curve was determined using pure L-proline (Merck KGaA, Hamburg, Germany) as the standard reference.

Superoxide dismutase (SOD) and catalase (CAT) activities were determined as described previously (Qiu et al., 2008). SOD activity was estimated spectrophotometrically as the inhibition of the nitroblue tetrazolium (NBT) photochemical reduction at 560 nm. CAT activity was determined based on the decrease in the level of H_2_O_2_. Each sample comprising 0.2 g of roots tissues was homogenized in 2 ml of 50 mM ice-cold phosphate buffer (pH 7.8) containing 1 mM ethylenediaminetetraacetic acid (EDTA). The homogenate was centrifuged at 15,000 × *g* for 15 min at 4°C. The supernatant comprised an enzyme extract containing both SOD and CAT.

### The Glycyrrhizic Acid, Liquiritin, Indoleacetic Acid, Jasmonic Acid, and Total Flavonoid Contents of Roots

To determine the total flavonoid content, 0.02 g of root powder was placed in 25 ml of 50% methanol for the extraction, followed by its ultrasonication at room temperature for 1.5 h; then, it was filtered and diluted to 25-ml volumetric flask, and set aside. Absorption measurements of these extracts for the determination of total flavonoids content were taken at 530 nm, using a UV–vis spectrophotometer and quantified with respect to the standard curve (Zou et al., 2012). The high-performance liquid chromatography (HPLC) analysis was conducted for GA, LIQ, indoleacetic acid (IAA), and JA; all extracts were dissolved in a small volume of 70% ethyl alcohol and filtered through a 0.22-μm microporous membrane. A 20-μl aliquot of each sample extract was analyzed, at 25°C, by high-performance liquid chromatography (HPLC) (Agilent1260 Infinity II, CA, United States). The GA and LIQ were measured as described previously; the detected wavelength was 254 nm (17.340 and 10.817 min, respectively) (Zheng et al., 2013). The IAA and JA were detected at 254 nm (14.900 min) and 210 nm (10.117 min), respectively.

### Metabolomic Analysis

All metabolite profiling analyses were performed independently at the Metware Biotechnology Co., Ltd. (Beijing, China). To do this, the company followed a standardized protocol [metabolites extraction, liquid chromatography–tandem mass spectrometry (LC-MS/MS) analysis, data preprocessing and annotation] exactly as described in Smith et al. (2006), Dunn et al. (2011), Kuhl et al. (2012), and Wang et al. (2016). Full details on the methodology used by that company can be found in Supplementary Material 1.

### Statistical Analysis

All experimental data were analyzed by ANOVA using SPSS 17.0 software (SPSS Inc., Chicago, IL, United States). After a significant *F*-value, differences between means were assessed on a pairwise basis using the Tukey’s honestly significant difference (HSD), at *p* < 0.05. Mean values and their standard errors (SE) are presented.

## Results

### *Bacillus amyloliquefaciens* FZB42 Enhances Plant Growth Under Drought

**FIGURE 1:**
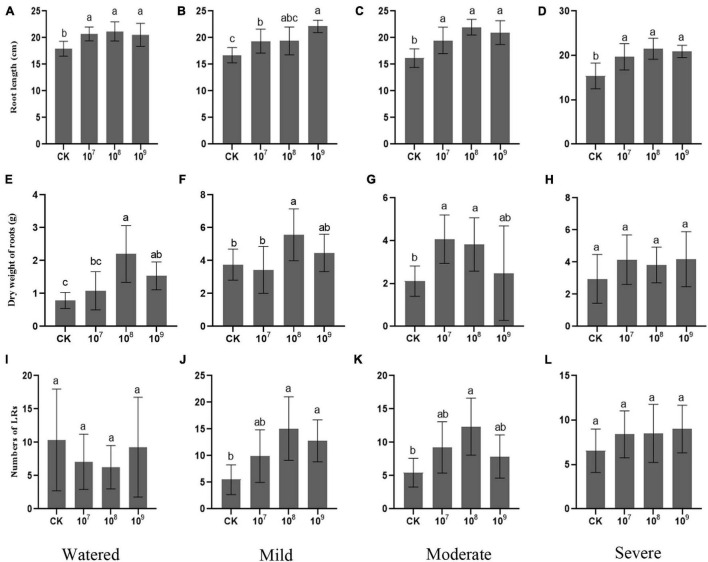
*Bacillus amyloliquefaciens* FZB42 improves the root growth of *Glycyrrhiza uralensis* under drought. The far-left column is the control group (watered condition) followed to the right by increasing drought stress conditions (mild, moderate, severe). The panel rows show growth responses in terms of **(A–D)** root length (*F*-value = 8.1, 9.6, 15.5, 11.5), **(E–H)** dry weight of roots (*F*-value = 13.8, 8.0, 5.9, 1.4), and **(I–L)** counts of lateral roots (LRs) (*F*-value = 1.1, 8.3, 7.4, 1.4). Bars are the mean ± SE (*n* = 15). CK, zero inoculum.

Under the watered condition, varied concentrations of FZB42 differentially improved both shoot and root growth. When inoculated with 10^8^ CFU ml^–1^, the root biomass increased substantially, to 2.5 times that of CK (control: zero inoculum), with a 16.9% longer root length. Although not as pronounced for root length, both biomass were also significantly promoted by the inoculation with FZB42 at a concentration of 10^9^ CFU ml^–1^. However, applying 10^7^ CFU ml^–1^ FZB42 can only significantly improve the root length but not biomass (Figures 1A,E). These results indicated that, under drought-free conditions, initial root inoculation with FZB42 improved the growth of *G. uralensis* roots for all three tested concentrations, but that of 10^8^ CFU ml^–1^ FZB42 was optimal. Moreover, this root promotion effect mainly impacted the axial root, where the numbers of lateral roots were similar irrespective of the inoculation treatments (Figure 1I).

Drought significantly affected the growth of *G. uralensis*, reducing this plant’s biomass (Figure 1). However, administering 10^8^ CFU ml^–1^ of FZB42 was beneficial to *G. uralensis*, resulting in this plant’s higher biomass accumulation (Figures 1B–D) and a longer root elongation under the three drought stress conditions while also enabling it to form more lateral roots (LRs) under the mild and moderate drought conditions (Figures 1J,K). Hence, these results indicated that 10^8^ CFU ml^–1^ FZB42 promoted the development of both axial and lateral roots in drought-stressed *G. uralensis*. The inocula of 10^7^ and 10^9^ CFU ml^–1^ FZB42 significantly promoted root elongation under all drought conditions, yet 10^7^ CFU ml^–1^ FZB42 was more effective for biomass accumulation under moderate and severe drought conditions, while using 10^9^ CFU ml^–1^ FZB42 did not increase the biomass in any drought treatment (Figures 1B–D,F–H). Taken together, 10^8^ CFU ml^–1^ FZB42 is evidently optimal for root growth promotion in *G. uralensis* under either normal (watered) or drought conditions. Under the watered condition, the FZB42 could only stimulate root’s axial elongation, but the bacterial strain was able stimulate both axial and lateral root growth in drought-stressed plants.

### *Bacillus amyloliquefaciens* FZB42 Improves the Accumulation Osmotic Adjustment Solutes and Antioxidase Activity in *Glycyrrhiza uralensis*

**FIGURE 2:**
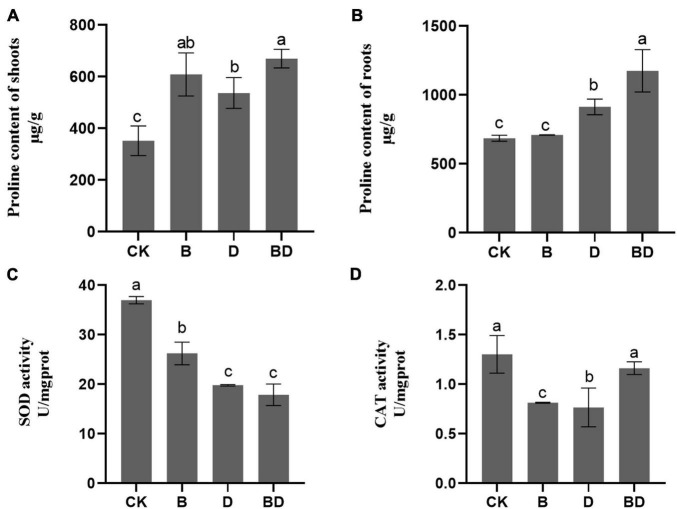
Plant osmotic adjustment solutes’ content and antioxidase activities. The proline content of **(A)** shoot and **(B)** root tissues, and the **(C)** SOD and **(D)** CAT activities of *Glycyrrhiza uralensis* (*F*-value = 15.1, 24.1, 85.9, 10.5). Bars are the mean ± SE (*n* = 3).

These analyses focused on the samples from plants inoculated with 10^8^ CFU ml^–1^ given its strong positive effects on root growth and drought tolerance. Here, we abbreviated the 10^8^ CFU ml^–1^ FZB42 under the normal watering condition treatment as “B” (i.e., 10^8^ CFU ml^–1^ FZB42 applied, with zero drought stress to plants) and, conversely, moderate drought without the FZB42 treatment as “D” (i.e., 0 CFU ml^–1^ FZB42 applied, with drought stress to plants). Correspondingly, to infer the joint effect, 10^8^ CFU ml^–1^ FZB42 under moderate drought treatment was designated as “BD” (i.e., 10^8^ CFU ml^–1^ FZB42 applied, with drought stress to plants). Hence, treatment B, D, and BD values could be compared to each other, as well to the CK (i.e., 0 CFU ml^–1^ FZB42 applied, with zero drought stress to plants). The proline content of both the shoot and root parts were generally increased by drought (Figure 2). However, these proline levels were significantly higher in BD than D at 3 months post-treatment. These results suggested that the FZB42 inoculation fostered proline accumulation in *G. uralensis* plant tissues, above and belowground, so as to restore their cell osmotic equilibrium disrupted by water deficits.

Two months after FZB42 inoculation, we found markedly decreased SOD and CAT activities under the watered condition, with no significant difference detected in SOD between D and BD. Yet intriguingly, a significant increase in CAT activity occurred in BD compared with B, which indicated that the bacterial strained improved oxidation resistance of *G. uralensis* by enhancing the antioxidant enzyme CAT’s activity in the roots.

### Multivariate Analysis and Identified Metabolites

**FIGURE 3:**
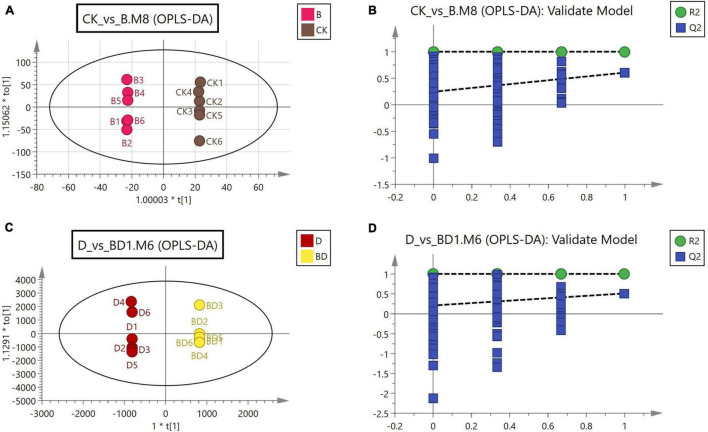
Score scatterplots of OPLS-DA **(A,C)** and validation plots **(B,D)** for two comparative groupings: CK vs. B, and D vs. DB.

To further investigate the effects of FZB42 inoculation on metabolism in *G. uralensis*, we used the CK, B, D, and BD treatment combinations in the metabolomic analysis. The Orthogonal Projections to Latent Structures Discriminant Analysis (OPLS-DA) models were used to analyze the metabolome data in the data matrix built. For the two comparative groupings of CK vs. B and B vs. BD, their score plots from the fitted OPLS-DA models and corresponding validation plots were constructed (Figure 3). To screen for differentially expressed metabolites in roots of either comparative grouping, we integrated the results of the multivariate and univariate to obtain suitable criteria for their designation: VIP > 1 for the first principal component in the OPLS-DA, *p*-value < 0.05, and fold-change (FC) > 1. Overall, 1,811 metabolites were identified, including 13 up- and 15 downregulated metabolites in the CK vs. B grouping. In the D vs. BD grouping, 79 metabolites were upregulated, while 24 were downregulated. Therefore, differential metabolites in the roots of *G. uralensis* with/without FZBZ42 inoculation under normal or drought conditions were identified.

### Kyoto Encyclopedia of Genes and Genomes Functional Annotation and Enrichment Analysis of Differential Metabolites

**FIGURE 4:**
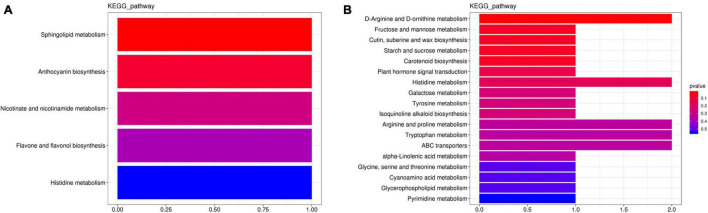
Kyoto Encyclopedia of Genes and Genomes pathways’ enrichment for **(A)** the CK vs. B comparative grouping and **(B)** the D vs. DB comparative grouping.

Differential expressed metabolites were mapped using the Kyoto Encyclopedia of Genes and Genomes (KEGG) database onto the KEGG pathways, for which those with increased or decreased differential metabolites (and enzymes) are presented in Figure 4. By comparing the metabolites of roots between CK and B, they could be mapped to five KEGG pathways and likewise to 22 KEGG pathways for those found between D and BD. In the CK vs. B comparative grouping, sphingolipid metabolism (ko00600), anthocyanin biosynthesis (ko00942), nicotinate and nicotinamide metabolism (ko00760), and flavone and flavonol biosynthesis (ko00944) all featured positive enrichment, whereas histidine metabolism (ko00340) underwent a negative enrichment (Figure 4A). In the D vs. BD comparative grouping, ABC transporters (ko00240), starch and sucrose metabolism (ko00500), fructose and mannose metabolism (ko00051), arginine and proline metabolism (ko00330), D-arginine and D-ornithine metabolism (ko00472), tryptophan metabolism (ko000380), and histidine metabolism (ko00340) were all significantly upregulated, while cutin, suberine, and wax biosynthesis (ko00073); carotenoid biosynthesis (ko00906); and plant hormone signal transduction (ko04075) were each downregulated (Figure 4B).

These results showed that FZB42 inoculation enhanced the biosynthesis of anthocyanin, which functions to mitigate stress in plants by ameliorating oxidative damage, as demonstrated in *Arabidopsis* and tomato (Tohge and Fernie, 2017). In addition, nicotinate and nicotinamide metabolism are positively related to nicotinamide adenine dinucleotide (NAD^+^), a redox carrier and a signal molecule. Accordingly, we speculate that FZB42 contributes to maintaining sufficient NAD^+^ pools in *G. uralensis*, which could protect this plant’s cells from energy depletion caused by adverse environmental conditions, thereby avoiding cell death and plant productivity loss as posited by Gakiere et al. (2018). Altogether, our results suggested the FZB42 treatment (10^8^ CFU ml^–1^) positively enriched proline and sucrose synthesis under moderate drought stress.

### *Bacillus amyloliquefaciens* FZB42 Augmented the Indoleacetic Acid and Jasmonic Acid Contents of Roots to Improve the Drought Stress Tolerance of *Glycyrrhiza uralensis*

For the metabolism of tryptophan, the precursor of IAA, it was positively enriched after FZB42 under the drought condition (Figure 4B), and the IAA content of the root tissue was determined by HPLC. Compared with the CK, the IAA content of plants that received the FZB42 treatment B decreased significantly under the watered condition, and conversely, it increased under the moderate drought condition. IAA accumulation might thus be an essential factor for sustaining root and shoot biomass production; stated differently, the mechanism by which FZB42 promoted plant growth under drought stress could involve the maintenance of IAA biosynthesis.

Furthermore, FZB42 inoculation increased the JA content of *G. uralensis* roots, under both watered and drought conditions. This result is consistent with the findings of similar studies on *Arabidopsis* previously reported (Hao et al., 2016; Liu et al., 2017; Lu et al., 2018; Liu S. et al., 2020). Hence, FZB42 may likewise activate the JA pathway in *G. uralensis*.

### *Bacillus amyloliquefaciens* FZB42 Improves Secondary Metabolites’ Accumulation Under Drought Stress

The positive enrichment of flavone and flavonol biosynthesis (ko00944) suggests that FZB42 is able to promote flavonol biosynthesis in *G. uralensis* under the normal condition (Figure 4A). The GA, LIQ, and total flavonoid content were determined to confirm this speculation.

**FIGURE 5:**
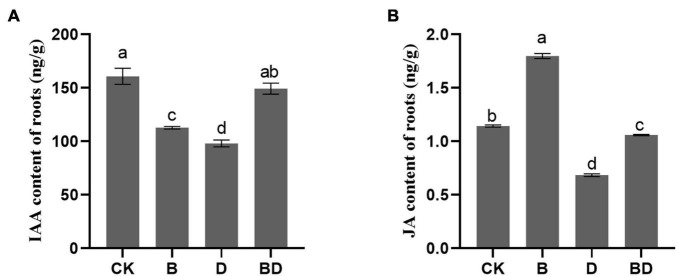
Phytohormones in the roots of *Glycyrrhiza uralensis*. The **(A)** indoleacetic acid content and **(B)** jasmonic acid content (*F*-value = 109.9, 3,153.8). Bars are the mean ± SE (*n* = 3).

Regarding GA and LIQ, their contents were generally decreased after the plants were subjected to drought stress, which indicated that the accumulation of secondary metabolites would be inhibited when plants were subjected to long-term moderate drought. Compared with CK, the FZB42 inoculation treatment (B) diminished the GA and LIQ contents but increased that of its total flavonoid content. This demonstrated that FZB42 can improve flavonoids’ biosynthesis, corroborating the results for the metabolites analysis, but did not enable GA and LIQ to accumulate (Figures 5A,C). However, the contents of GA, LIQ, and total flavonoids were significantly higher in BD than D and on par with those of B, which suggested that the FZB42 inoculation seems to limit losses of GA and LIQ accumulation in plants caused by drought stress (Figure 5). Interestingly, we uncovered a strong positive correlation between total flavonoids content and the JA content in roots (Pearson’s *r* = 0.629, *p* = 0.028), which pointed to the JA pathway’s involvement in the flavonoids’ accumulation in *G. uralensis*.

## Discussion

### Effect of *Bacillus amyloliquefaciens* Upon Plant Growth

Water is essential for plant survival, and chronic water deficits constrain plant growth and fitness (Gupta et al., 2020). Studies have shown that inoculations with PGPR could improve drought stress tolerance of various plant species (Chanway and Holl, 1994; Saravanakumar et al., 2010; Marasco et al., 2012). In this study, the drought tolerance ability of *G. uralensis* was significantly enhanced after inoculating its rhizosphere with the strain *B. amyloliquefaciens* FZB42; specifically, it tempered the drought-induced reductions the length of roots, the number of lateral roots, and the dry weight of root tissue (Figure 1). This result is consistent with several previous studies (Kang et al., 2013; Tiwari et al., 2016; Xie et al., 2019). Greater water and nutrition acquisition by roots is crucial for sustaining plants’ performance in the face of drought stress. Auxin plays an important role in the molecular mechanisms of action of PGPR on root architecture, which including directly synthesis from tryptophan in plant root exudates and indirectly activated by various signaling chemicals (Kalyanasundaram et al., 2021). Therefore, our results provide strong evidence that inoculation with *B. amyloliquefaciens* could play an important role in improving the growth and biomass of *G. uralensis* through promoting root growth and altering root architecture under drought stress conditions.

### Effect of *Bacillus amyloliquefaciens* on Tryptophan Metabolism and Indoleacetic Acid’s Accumulation

**FIGURE 6:**
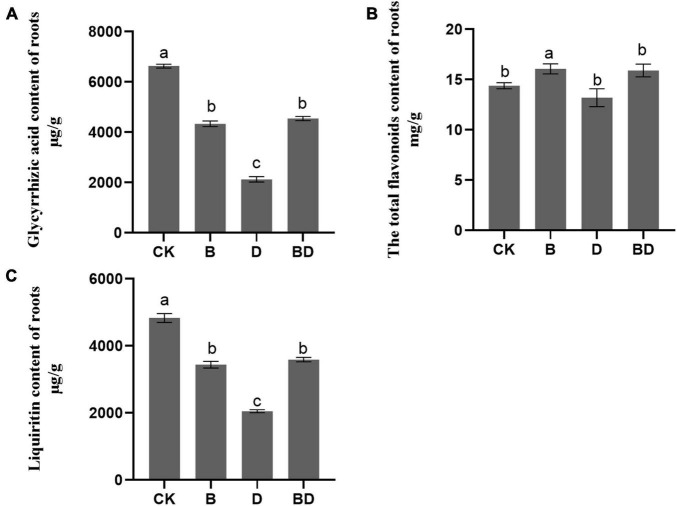
Secondary metabolites of *Glycyrrhiza uralensis*. The **(A)** GA content, **(B)** total flavonoids content, and **(C)** LIQ content of roots (*F*-value = 1,114.8, 465.8, 14.5). Bars are the mean ± SE (*n* = 3).

Plant hormones are among the most critical growth regulators. The plant growth and stress tolerance induced by beneficial microorganisms partly depends on their ability to facilitate synthesis of phytohormones in the rhizosphere or root tissue network (Davies et al., 1996; Bano et al., 2013). Being the principal auxin naturally occurring in most plants, IAA figures prominently in many key biological processes, including cell division, elongation, differentiation, and leaf expansion (Estelle, 1992). Furthermore, higher levels of IAA are expected to lower intracellular ROS levels (Guan and Scandalios, 2002; Fei et al., 2016; Khaksar et al., 2017). A work by Ma et al. (2011) highlighted the positive role of plant-exuded IAA in activating those bacterial genes responsible for the colonization of host plants’ roots and adaptation to them. For example, the *Bacillus subtilis* strain GB03 promotes *Arabidopsis* growth *via* upregulating transcripts for auxin homeostasis (Zheng et al., 2013). Tryptophan is the precursor of IAA; we found that tryptophan metabolism (ko000380) upregulated, along with the content of IAA augmented after FZB42 inoculation of *G. uralensis* plants under drought stress (Figure 6). In addition, analogously to other PGPR, FZB42 owns the ability of IAA production that might have positive effects on root architecture alterations, which could promote plant growth as a whole (Helmut and Rainer, 2004). The IAA accumulation results in more root tips and a larger root surface area (Figure 1), thereby enabling greater water and nutrient acquisition under drought conditions. In sum, FZB42 inoculation induced IAA accumulation, which can ensure the growth of drought-stricken *G. uralensis* plants—all of which survived the experiment—and generally enhanced its drought tolerance.

### Effect of *Bacillus amyloliquefaciens* on the Osmolytes and Antioxidants *via* Activation of the Jasmonic Acid Pathway

Osmotic adjustment is one of the key adaptations at the cellular level that helps plants tolerate drought-induced oxidative damage (Farooq et al., 2009; Huang et al., 2014). Under abiotic stress, plant accumulate solutes, including sugars (e.g., sucrose) and non-protein amino acids (e.g., proline), to maintain cellular turgor and help plants lower water potential without decreasing actual water content (Farooq et al., 2008). Proline acts as the key role for osmotic adjustment and also contributes to stabilizing subcellular structures, scavenging free radicals, and buffering cellular redox potential (Hayat et al., 2012). Treatment of plants with PGPR has been shown to increase proline levels (Casanovas et al., 2002; Sziderics et al., 2007; Bano et al., 2013; Sharma et al., 2013; Gusain et al., 2015). In accordance with this, the metabolome analysis and proline content results confirmed that inoculation with FZB42, a PGPR, induced proline accumulation under drought condition (Figures 2B, 4B). In addition, our metabolomics results showed that soluble sugars-related metabolism, starch and sucrose metabolism (ko00500), and fructose and mannose metabolism (ko00051) were upregulated in plant roots (Figure 4B). During water loss, soluble sugars function critically as osmoprotectants in maintaining turgor pressure with a sufficient degree of hydration (Loutfy et al., 2011; Krasensky and Jonak, 2012). Moreover, we found that the FZB42 treatment also enhanced the accumulation of starch, which is directly linked to the photosynthetic capacity of plants under drought stress (Sharma et al., 2020). We, thus, may reasonably infer that FZB42 inoculation could induce a plant’s tolerance of drought stress by modifying its osmotic adjustment.

Furthermore, plants can increase the activities of certain antioxidant enzymes, namely, CAT, SOD, GPX, APX, and GR, to clear out H_2_O_2_ and O_2_^–^ from their cells (Tian et al., 2012). Because SOD converts O_2_^–^ to H_2_O_2_, it is thought to play a fundamental role in the antioxidant defense system, while CAT and POD destroy H_2_O_2_ in the cytoplasm and other cell components. In previous studies, drought stress altered the amount and activities of enzymes involved in scavenging for ROS in various species (Sankar et al., 2007; Uzilday et al., 2012; Akitha Devi and Giridhar, 2013). In our study, inoculation with *B. amyloliquefaciens* FZB42 was capable of increasing the activity of CAT, but that of SOD did not change much (Figure 2). These results suggest that the improved CAT was induced by PGPR *B. amyloliquefaciens* to remove H_2_O_2_ and produce H_2_O, thereby keeping free radicals at a lower abundance and avoiding lipid peroxidation damage of cell membrane in *G. uralensis*. This is similar to findings of previous studies in basil (*Ocimum basilicum* L.), maize (*Zea mays* L.), and tomato (Heidari and Golpayegani, 2012; Naveed et al., 2014).

Jasmonic acid can contribute to tolerance of drought stress in plants by increasing the activity of their antioxidants (Bao et al., 2009). In a recent study, methylated JA (MeJA) was found able to alleviate oxidative stress caused by salt stress in *G. uralensis* seedlings *via* enhancing the activity of antioxidant enzymes and non-enzymatic antioxidants (Lang et al., 2020). PGPR was considering to have potential to enhance plant growth directly by improving nutrition, regulating stress phytohormone like JA, increasing antioxidants activity, and producing siderophores (Bukhat et al., 2020). Kang et al. (2014) reported that *P. putida* H-2-3 modulates JA and antioxidants expression of soybean to improve the plant growth under saline and drought conditions. Given that FZB42 induced JA’s accumulation under drought stress (Figure 6), we speculate that both this PGPR strain and its volatile organic compounds (VOCs) were able to activate the JA pathway to defend against this abiotic stress, which is consistent with our previous work (Liu et al., 2017; Liu S. et al., 2020).

### *Bacillus amyloliquefaciens* Affects the Production of Secondary Metabolites by Activating the Jasmonic Acid Pathway

Although moderate drought stress is reported to show beneficial effect on secondary metabolites accumulation in medicinal plants (Bloem et al., 2014), a previous study showed that the contents of total flavonoids, total polysaccharides, and glycyrrhizic acid were significantly decreased by drought stress (Xie et al., 2019). *Bacillus pumilus* inoculation could alleviate the abiotic-stress-induced negative effects on the secondary metabolite accumulation in *Mentha arvensis* and *G. uralensis* (Bharti et al., 2013; Xie et al., 2019). In this study, we found that FZB42 inoculation could interfere with the suppression of total flavonoid content and LIQ (liquiritin) and GA (glycyrrhizic acid) contents caused by drought stress (Figure 5).

Jasmonic acid and its related compounds are considered as transducers of elicitor signals for plant secondary metabolites (Devoto et al., 2005; Zhai et al., 2017). The role of MeJA in the secondary metabolites of *G. uralensis* has been considerable recently. Treatment with MeJA increased the GA content of *G. uralensis* but limited its root growth (Shabani et al., 2009; Li et al., 2016). In another experiment, the MeJA treatment spurred the production of total flavonoids in a suspension of cultured cells of the conspecific plant, *G. inflata* (Ying et al., 2008). In addition, MeJA improved metabolism and sucrose content of NaCl-stressed *G. uralensis* seedlings (Lang et al., 2020). Thus, MeJA exerts a beneficial effect on secondary metabolites production of *G. uralensis*. In addition, JA combined with other secondary messengers was reported to regulate the synthesis of secondary metabolites *via* microbial elicitors (Hu et al., 2006). Moreover, the increased sucrose content reportedly caused by JA rescued the GA loss caused by salt condition in *G. uralensis* (Fuzhi and Jun, 2015; Lang et al., 2020). Based on these results, we suppose that FZB42 activated JA pathway and promoted JA accumulation in *G. uralensis*; these excess JAs further enhanced the secondary metabolites accumulation. Meanwhile, the diminished root growth caused by JA was partly rescued by the accumulation of IAA.

## Conclusion

**FIGURE 7:**
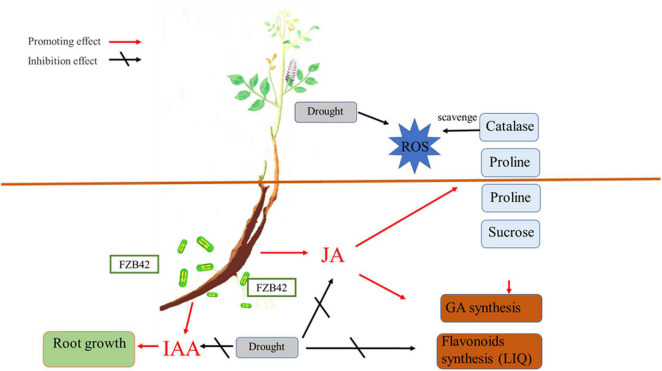
Postulated mechanism by which the *Bacillus amyloliquefaciens* FZB42 strain induces drought tolerance and rescues glycyrrhizic acid (GA) loss in drought-stressed *Glycyrrhiza uralensis* plants *via* the JA pathway’s activation. IAA, indoleacetic acid; JA, jasmonic acid; ROS, reactive oxygen species.

In conclusion, our experimental results demonstrate that the drought stress tolerance of *G. uralensis* can be improved by inoculation with *B. amyloliquefaciens* FZB42 by modifying root architecture and increasing the antioxidant enzyme activities and proline and sucrose contents. *Bacillus amyloliquefaciens* FZB42 can also rescue the GA, LIQ, and total flavonoid losses caused by drought. The greater JA content, a consequence of FZB42 inoculation, likely fosters favorable physiological changes in plants under drought stress. These results suggest that inoculation with *B. amyloliquefaciens* strains may offer a promising and practical way to improve the drought tolerance of cultivated *G. uralensis* plants (summarized in Figure 7). Our results demonstrated that inoculation of FZB42 showed both significant plant growth promotion and accumulation of GA, LIQ, and total flavonoid in *G. uralensis* under drought condition. It will guide a new strategy for cultivating the Chinese herbal plant *G. uralensis*.

## Data Availability Statement

The original contributions presented in the study are included in the article/Supplementary Material, further inquiries can be directed to the corresponding author.

## Author Contributions

LY and RW conceived and designed the experiments. LY and CU performed the experiments and wrote the manuscript. YT and YL analyzed the data. XZ, QZ, BL, ZC, and CD provided the technical assistance to LY. YW and YZ revised the language of the manuscript. All authors contributed to the article and approved the submitted version.

## Conflict of Interest

The authors declare that the research was conducted in the absence of any commercial or financial relationships that could be construed as a potential conflict of interest.

## Publisher’s Note

All claims expressed in this article are solely those of the authors and do not necessarily represent those of their affiliated organizations, or those of the publisher, the editors and the reviewers. Any product that may be evaluated in this article, or claim that may be made by its manufacturer, is not guaranteed or endorsed by the publisher.
