# Supplementary material for: Bacillus amyloliquefaciens Rescues Glycyrrhizic Acid Loss Under Drought Stress in Glycyrrhiza uralensis by Activating the Jasmonic Acid Pathway
Source: Front Microbiol. 2022 Mar 16;12:798525. doi: 10.3389/fmicb.2021.798525 (PMC8966401; doi:10.3389/fmicb.2021.798525)
Supplement: Supplementary file 1 [file Data_Sheet_1.docx]

Supplementary Material

**Metabolites extraction**

5 mg of sample was weighted to an EP tube, and 500 μL extract solution (acetonitrile: methanol: water = 2: 2: 1) containing isotopically-labelled internal standard mixture was added. After 30 s vortex, the samples were homogenized at 35 Hz for 4 min and sonicated for 5 min in ice-water bath. The homogenization and sonication cycle was repeated for 2 times. Then the samples were incubated at -40 ℃ for 1 h and centrifuged at 12000 rpm for 15 min at 4 ℃. 250 μL of supernatant was transferred to a fresh tube and dried in a vacuum concentrator at 37 ℃. Then, the dried samples were reconstituted in 300 μL of 50% acetonitrile by sonication on ice for 10 min. The constitution was then centrifuged at 13000 rpm for 15 min at 4 ℃, and 75 μL of supernatant was transferred to a fresh glass vial for LC/MS analysis. The quality control (QC) sample was prepared by mixing an equal aliquot of the supernatants from all of the samples.

**LC-MS/MS analysis**

The UHPLC separation was carried out using a 1290 Infinity series UHPLC System (Agilent Technologies), equipped with a UPLC BEH Amide column (2.1× 100 mm, 1.7 μm, Waters). The mobile phase consisted of 25 mmol/L ammonium acetate and 25 mmol/L ammonia hydroxide in water（pH = 9.75）(A) and acetonitrile (B). The analysis was carried out with elution gradient as follows: 0~0.5 min, 95%B; 0.5~7.0 min, 95%~65% B; 7.0~8.0 min, 65%~40% B; 8.0~9.0 min, 40% B; 9.0~9.1 min, 40%~95% B; 9.1~12.0 min, 95% B. The column temperature was 25℃. The auto-sampler temperature was 4 ℃, and the injection volume was 2 μL (pos) or 2 μL (neg), respectively.

The TripleTOF 6600 mass spectrometry (AB Sciex) was used for its ability to acquire MS/MS spectra on an information-dependent basis (IDA) during an LC/MS experiment. In this mode, the acquisition software (Analyst TF 1.7, AB Sciex) continuously evaluates the full scan survey MS data as it collects and triggers the acquisition of MS/MS spectra depending on preselected criteria. In each cycle, the most intensive 12 precursor ions with intensity above 100 were chosen for MS/MS at collision energy (CE) of 30 eV. The cycle time was 0.56 s. ESI source conditions were set as following: Gas 1 as 60 psi, Gas 2 as 60 psi, Curtain Gas as 35 psi, Source Temperature as 600 ℃, Declustering potential as 60 V, Ion Spray Voltage Floating (ISVF) as 5000 V or -4000 V in positive or negative modes, respectively.

**Data preprocessing and annotation**

MS raw data (.wiff) files were converted to the mzXML format by ProteoWizard, and processed by R package XCMS (version 3.2). The process includes peak deconvolution, alignment and integration. Minfrac and cut off are set as 0.5 and 0.3 respectively. In-house MS2 database was applied for metabolites identification.
